# Early experience with COVID-19 in kidney transplantation recipients: update and review

**DOI:** 10.1590/S1677-5538.IBJU.2020.S114

**Published:** 2020-07-27

**Authors:** Javier González, Gaetano Ciancio

**Affiliations:** 1 Hospital General Universitario Gregorio Marañón Servicio de Urología Madrid España Servicio de Urología. Hospital General Universitario Gregorio Marañón, Madrid, España; 2 University of Miami Miller School of Medicine Department of Surgery MiamiFL US Department of Surgery, University of Miami Miller School of Medicine, Miami, FL, US; 3 University of Miami Miller School of Medicine Department of Urology MiamiFL US Department of Urology, University of Miami Miller School of Medicine, Miami, FL, US; 4 Miami Transplant Institute Jackson Memorial Hospital MiamiFL US Miami Transplant Institute, Jackson Memorial Hospital, Miami, FL, US

**Keywords:** Infections, COVID-19 [Supplementary Concept], Immunosuppression, Kidney Transplantation

## Abstract

**Introduction:**

little is known on the risk factors, clinical presentation, therapeutic protocols, and outcomes of kidney transplantation recipients (KTRs) who become infected by SARS-CoV-2.

**Purpose:**

to provide an updated view regarding the early experience obtained from the management of KTRs with COVID-19.

**Materials and Methods:**

A narrative review was conducted using PubMed database to identify relevant articles written in English/Spanish, and published through May 15, 2020. Search terms included: “coronavirus”, “severe acute respiratory syndrome coronavirus 2”, “SARS-CoV-2”, “COVID-19”, “COVID”, “renal transplantation”, and “kidney transplantation”. Case series were considered eligible, and case reports excluded. Thirty-four articles were included in the review.

**Results:**

KTRs should be considered immunocompromised hosts: potential risk for infection, non-negligible comorbidity, and exposure to long-term immunosuppression. Only single center small retrospective experiences are still available regarding KTRs with COVID-19. SARS-CoV-2 symptoms in KTRs are similar to that observed for the general population, being fever and cough the most frequently observed. Mild-to-moderate symptomatic KTRs can be managed in an outpatient setting, while patients exhibiting severe symptoms must be addmited to hospital. More rapid clinical progression, and higher complication and death rates have been observed for hospitalized KTRs, requiring hemodyalisis or ventilatory support. Lymphopenia, elevated serum markers (C-reactive protein, procalcitonin, IL-6, D-dimer), and chest-X-ray findings consistent with pneumonia are linked to worse prognosis. A number of antiviral therapies have been used. However, it is difficult to draw meaningful conclusions regarding their efficacy at this point. Baseline immunosupression regimen should be adjusted in a case-by-case manner. However, it poses a significant challenge.

## INTRODUCTION

Since december 2019, a growing number of atypical pneumonia cases of unknown origin were initially detected in different medical centers of Wuhan (Hubei, China). The infection spread rapidly across the World causing a global pandemic in only three months (1). The analysis of the genome sequence of specimens retrieved from the respiratory tract of those patients, revealed a single-stranded and positive-sense RNA virus as etiological agent. This virus share close similarities in its structure with the severe respiratory syndrome coronavirus (SARS-CoV) that cause the SARS global pandemic in 2003, and the Middle East respiratory syndrome (MERS) epidemic in 2012 (MERS-CoV) (2–4). The novel coronavirus was so-called severe acute respiratory syndrome coronavirus 2 (SARS-CoV-2) by the International Comitee on Taxonomy of Viruses. The disease produced by SARS-CoV-2 was named Coronavirus Disease 2019 (COVID-19) by World Health Organization (5), after declaring it a potentially lethal infectious disease posing a real threat to global health security, as evidenced by a dramatic total of 4,466,944 new cases, and 299,507 deaths by May 15th, 2020 since the beginning of the pandemic worldwide (6).

Although the clinical debut resembles that produced by other common respiratory viruses, the course may evolve to a potentially life-threatening respiratory distress, multi-organ failure, or even death in a short time frame. The infection may cause other disorders affecting mainly the gastrointestinal and nervous systems. It has been reported to affect more severely to older patients, and those exhibiting a number of comorbid conditions including hypertension (6%), diabetes (7.3%), immunosuppresion, lung or cardiac insufficiency (10.5%), chronic kidney disease (CKD), cancer (5.6%), and renal replacement therapy (RRT) (7).

Kidney transplantation recipients (KTRs) should be considered immunocompromised hosts for their unique potential risk for COVID-19 infection, given their non-negligible comorbidity, exposure to long-term immunosuppression, and residual CKD. In fact, the SARS pandemic was reported to affect KTRs (8), and various solid organ transplantation recipients died in both SARS and MERS epidemics (9, 10). However, to date little is known on the risk factors, clinical presentation, diagnostic troubles, therapeutic protocols, and outcomes of KTRs who become infected by SARS-CoV-2. The aim of this review is to provide an updated view regarding the early experience obtained from their management.

## MATERIALS AND METHODS

A literature review was conducted using PubMed database to identify relevant articles written in English or Spanish, and published through May 15, 2020. Search terms included “coronavirus”, “severe acute respiratory syndrome coronavirus 2”, “SARS-CoV-2”, “COVID-19”, “COVID”, “renal transplantation”, and “kidney transplantation”. Due to the lack of randomized controlled trials, case series were considered eligible for inclusion. Case reports were excluded. The initial search provided 45 articles, which abstracts were independently reviewed. Finally, 34 articles reporting on KTRs with SARS-CoV-2 infection were reviewed.

### Comorbid conditions and treatment

KTRs are at a higher risk to COVID-19 infection due to immunosuppression, underlying CKD, and other comorbid conditions, in particular hypertension (HTN) and diabetes (DM) (11). However, important comorbidity is inherent to CKD and RRT, thus being quite common in a KTR. Table-1 includes the most representative series reviewed (9 series; N=184 patients), summarizing all the relevant information regarding demographics, transplantation, manteinance immunosuppression regimen, and comorbid conditions of the patients included. Most of these patients (15-94%) exhibited at least one comorbid condition such as HTN (40-100%), DM (15-69%), active cancer (3-20%), and chronic heart or lung disease (15-42%).

**Table 1:** Summary of demographics, comorbid conditions, time from transplantation, source of donation, and baseline immunossuppression regimen.

Author	#Number of patients	Age range (yrs.)	Gender Μ/F (%)	Comorbid conditions (%)	Time from Transplant range (months)	Source of donation DD/LD (%)	Baseline immunosuppression regime				
							Anti-Mb (%)	CNI (%)	m-TOR I (%)	GC (%)	AB (%)
Banerjee D, et al. (14)	7	45-69	57/43	HTN 85	1-360 (28% first 3 months)	100/0	100	85	0	71	0
				DM 42							
				O: 42							
Alberici, et al, (7)	20	41-73	80/20	HTN: 85	108-240	N/A	70	95	10	65	0
				DM: 15							
				O: 15							
CUKTP (1)	15	28-72	66/33	N/A	38-118	80/20	86	93	0	67	13
Zhang, et al, (24)	5	37-64	80/20	HTN:40	2-36	100/0	80	80	20	80	0
				DM:20							
				Cancer:20							
Pereira, et al, (16)	48 (2% kidney-pancreas, 2% liver-kidney)	46-68	53/47	HTN: 64	35-127	N/A	76	86	7	59	3
				DM: 46							
				Cancer: 3							
				O: 20							
									
Akalin, et al, (13)	36	32-77	72/28	HTN: 94	N/A	75/25	86	97	0	94	0
				DM:69							
				0: 17							
Zhu, et al, (15)	10	24-65	80/20	HTN: 50	6-144	7/3	100	100	0	70	0
				O: 30							
Montagud-Marrahi, et al, (18)	33 (6% kidney-pancreas)	40-74	58/42	N/A	48-180	N/A	62,5	87,8	42,4	78,8	0
									
									
Nair, et al, (23)	10	47-67	60/40	HTN:100	N/A	50/50	100	90	10	70	6
				DM: 90							

**CUKTP** = Columbia University Kidney Transplant Program; **M** = male; **F** = female; **DD** = deceased donor; **L/D** = living donor; **Anti-Mb** = antimetabolite therapy; **CNI** = calcineurin inhibitors; **m-TOR I** = m-TOR inhibitors; **GC** = glucocorticoid therapy; **AB** = monoclonal/polyclonal antibodies; **HTN** = hypertension; **DM** = diabetes mellitus; **0** = others (including heart or lung chonic disease, HIV infection, HCV infection, CMV infection, and hemolytic anemia)

They were receiving a number of medications for comorbidity control (i.e., mainly a wide variety of antihypertensives, antidiabetics, and statins). It has been hypothesized that SARS- -CoV-2 uses angiotensin-converting enzyme 2 (ACE2) to gain entry in the cells, making ACE inhibitors and/or angiotensin receptor blockers (ARBs) increase the risk of SARS-CoV-2 pneumonia via altered expression of ACE2. There are no clinical data in favor or against this hypothesis, and changing the doses of ACE inhibitors/ARBs during the treatment of infection seems not recommended (12). Similarly, no recommendation is advised regarding the remaining concomitant therapy. It seems prudent keeping the current medication inaltered unless otherwise specified, and act in a case-by-case basis according to the situation exhibited by a particular patient.

### COVID-19 infection

SARS-CoV-2 causes a variety of symptoms including upper respiratory (sore throat), lower respiratory (cough and dyspnea), constitutional (fever, malaise, myalgia), gastrointestinal (nausea, vomiting, abdominal pain, diarrhea), or a combination of them. Many patients have also reported anosmia or dysgeusia; somewhat a unique feature of this syndrome (13). COVID-19 symptoms were reported frequently among the patients included. Fever (58-100%) and cough (42-100%) were noted almost invariably, followed by diarrhea (20-90%), dyspnea (5-90%), fatigue or myalgia (5-90%), and coryza (10%), similar to that observed for the general population (Table-2). Interestingly, no neurologic symptoms were recorded.

**Table 2:** Summary of symptomatology, diagnostic test findings, and outcomes.

Author	Clinical presentation (symptom %)	Blood parameters (present/absent moderate <50% patients, intense>50% patients)	PCR test (%)	CXR (%)	Sat02 <93% (%)	Complic, Rate (%)	Outcome					Definitive outcome (%)
							Outpatient rate (%)	Hosp Adm (%)	ICU Adm (%)	Death rate (%)	Discharge rate (%)	
Banerjee D, et al, CM)	Fever(*) 71, Cough 42, Dyspnea 57, Myalgia 14	Lymphopenia, Intense elevation of CRP, D-dimer, LDH, ESR	100	100	57	ARDS 42 AKI 28 TE 14 Sepsis 14	29	71	57	14	14	57
Alberici, et al, (7)	Fever (*)100, Cough 50, Myalgia 5, Dyspnea 5	Moderate elevation of LDH, Urea and Cr Intense elevation of CRP, procalcitonin, ferritin, D-dimer	100	85	84	N/A	o	100	20	15	15	40
CUKTP (1)	Fever (*) 87, Cough 60, Myalgia 13 Diarhea 20	Lymphopenia, Moderate elevation of LDH, Intense elevation of CRP, ferritin, I-Troponin, ESR, IL-6	100	73	N/A	AKI 40	o	100	27	13	53	66
Zhang, et al, (24)	Fever (*)100, Cough (*)100, Myalgia 60	Lymphopenia, Moderate elevation of D-dimer and ESR, Intense elevation of CRP	100	100	N/A	N/A	o	100	o	o	40	40
Pereira, et al, (16)	Fever(*)70 Cough 59 Dyspnea 43 Myalgia 24, Diarrhea 31	Lymphopenia, hipoalbuminemia, Moderate elevation of Cr, I-Troponin, D-dimer, ferritin Intense elevation of CRP, procalcitonin, ferritin, and IL-6	100	100	42	N/A	24	76	34	24	54	78
Akalin, et al, (13)	Fever (*) 58, Myalgia 22	Lymphopenia, thrombocytopenia, Moderate elevation of ferritin, CRP procalcitonin, and D-dimer	100	96	N/A	AKI 21	22	78	N/A	28	N/A	N/A
Zhu, et al, (15)	Fever(*)90 Cough (*) 90 Dyspnea (*) 90 Myalgia (*) 90 Diarrhea (*) 90	Lymphopenia Moderate elevation of Cr, moderate elevation of liver enzymes	100	100	N/A	AKI 60 RRT 10	0	100	N/A	10	N/A	90
Montagut-Marrahi, et al, (18)	N/A	N/A	100	N/A	N/A	N/A	0	79	52	6	N/A	87
Nair, et al, (23)	Fever(*) 70, diarrea 20, coryza 10	Lymphopenia, Moderate elevation of CRP and ferrtin	100	N/A	N/A	AKI 50 RRT 10	0	90	50	30	70	100

**PCR** = polymerase chain reaction-test (positive result); **CXR** = chest-X-ray (findings); **SatO**_2_ = Oxygen saturation; **Adm** = admission; **ARDS** = acute respiratory distress síndrome; **AKI** = acute kidney injury; **LDH** = lactate dehydrogenase; **CRP** = C-reactive protein; **Cr**: serum creatinine; **ESR** = erythrocyte sedimentation rate; **IL-6** = interleukin-6; **RRT** = renal replacement therapy

(*) most frequent presenting symptom

Two different phases can be drawn in the clinical course of COVID-19: a first phase (7-10 days) characterized by viral replication and cytopathic effect, and a second phase associated to hyperinflammation and high cytokine release (i.e., cytokine storm), and characterized by progressive lung involvement, and escalating needs of oxygen supplementation and/or ventilatory support (14). Fever preceded dry cough, dyspnea, and chest tightness by several days, but the intervals observed varied widely and tended to be longer among the series included in this review (3-21 days). It has been hypothesized that, on one hand the immunosuppression may provoque a delay in viral clearance, while on the other hand, this therapy may induce some protective effect to the occurrence of fatal critical pneumonia caused by the hyperimmune response (15). However, a more rapid clinical progression than the general population has been noted in COVID-19 KTRs (13). This fact is confirmed by the data extracted from the series regarding hospital and intensive care unit admissions (76-100% and 20-57%, respectively), and the comparative disproportion of patients managed in an outpatient basis (22-29%). A possible explanation for this fact may be a potential selection bias, since the vast majority of the patients sought care presumably for severe symptoms, were hospitalized and derived for ICU care accordingly, and were included within the 3-week period (range :6-45 days) of hospitalization follow-up conducted by most centers. This fact is in line with the observation provided by Pereira et al. (16), who affirm that hospitalized cohorts, particularly those presenting dyspnea, show higher rates of severe disease. Nonetheless, experience with other infections in kidney transplant recipients shows that potentially serious infections may have subtle or delayed presentations, that should be linked to more proactive approaches in the diagnostic evaluation and monitoring, and lower threshold for hospitalization (12).

Clinical classification of COVID-19 pneumonia includes mild, severe, and critical types (l5). The cytokine storm, and hyperinflammation pattern due to antiviral immune response has been disscused as the driver for severe respiratory symptomatology and acute respiratory distress syndrome (ARDS). Many patients included in this review (42-84%) exhibited oxygen saturation levels < 93% at some point during their admission, thus requiring respiratory support with oxygen supplementation (65-85%), non-invasive ventilation (10-41%), or mechanical ventilation (6-39%) depending on their particular situation and moment during the clinical course. The rates for mechanical ventilation observed among the series studied seems sensibly higher compared to that reported for the general population (39 vs. 15%) (1), but again a selection bias would explain this disproportion.

Similarly to what was observed in the SARS-CoV and MERS, the uptake of SARS-CoV-2 into the proximal tubular epithelium is a posible explanation for acute kidney injury (AKI) in COVID-19 patients. AKI has been reported in up to 15% and 29% of the overall, and critically ill COVID-19 patients in the general population (17). Variable degrees of proteinuria and hematuria have also been reported. However, the studies included in this review show a different reality regarding kidney transplant recipients. AKI has been observed in 21-60%, requiring aproximately 10% RRT. These findings may presumably be atributed to acute tubular necrosis. However, given the circumstances of demand for assistance, and risks associated to non-essential tests, no for-cause biopsy was performed in any center.

AKI would be present or subsequently developed in KTRs more frequently than in the general population. It has been observed that patients with triple manteinance inmmunosuppression schedules, and those that require immunossuppressive induction, or aggressive therapy with monoclonal/polyclonal antibodies for an ongoing acute rejection may experiment more severe COVID-19 symptoms, longer clinical course, and need for RRT. This observation would favor a transient reduction or cessation in some (or even all) the immunosuppressive agents used to avoid an infection worsening, and in turn a potentially increased risk for acute rejection.

In fact, acute rejection may play a role in some of the AKI cases observed, but an accurate diagnosis cannot be provided. Nevertheless, AKI and RRT seems to lead to worse prognosis and outcome, explaining in part the excess in mortality observed in the series included (up to 30%) in comparison to that of the general population (0.221%, depending on age) for the general population and the KTRs, respectively (18). In fact, death seems more likely to be produced by extrapulmonary complications (i.e., thrombosis, sepsis) rather than from severe pneumonia or ARDS, reinforcing the idea that these complications, although scarce in frequency carry devastating consequencies in the short-term (3-week period until discharge or fatal event) (14).

### COVID-19 diagnosis and follow-up protocol during admission

The initial diagnosis is currently based on at least one of the following: clinical suspicion, alterations in the blood sample analysis, and chest X-ray (CXR) findings. Suspicion should be confirmed by specific testing.

COVID-19 was uniformly confirmed (100%) by nucleic acid polymerase chain reaction (PCR)-testing of swab samples obtained from the nose and/or throat of the patients included. However, <10% false negative cases were detected. This fact is possible due to problems in the sampling techniques, variable viral load of the upper respiratory tract, and mutations of the virus gene. Repeated PCR-testing (whole genome viral sequencing) by experienced staff, along with blood SARS-CoV-2 antibody detection, may solve this problem and optimize diagnosis. In cases of limited access to tests, symptoms prevail. Any patient with history of recent exposure, or in the presence of suggesting symptoms must be always considered a candidate for testing, and managed as presumptively positive unless specified otherwise. In case of high suspicion and negative PCR-testing, a new test must be repeated after 48 hours, and the patient considered positive in the meanwhile (19).

Initial blood test has to include red and white cell blood counts, metabolic and liver function biochemical panels, coagulation parameters, erythocyte sedimentation rate (ESR), C-reactive protein (CRP), and procalcitonin (le). Serum levels of albumin, D-dimer, ferritin, Interleukine-6 (IL-6), and I-Troponin have been also reported of value in the initial diagnosis, and would serve to categorize the severity of the infection.

Lower lymphocite counts, elevated ESR and serum levels of CRP, procalcitonin, D-dimer, ferritin, IL-6, and I-troponin at any point of the clinical course were uniformly reported among the series studied (Table-2). The cause for lymphocite depletion remains unclear, although lymphocytes have been identified as a primary target of SARS-Cov-2 injury, and somehow may be considered a normal feature in those patients receiving immunosuppresion. However, a further drop in lymphocyte count beyond the baseline should suggest disease worsening, thus representing a prognostic factor for severe illness. Leukocyte and neutrophil counts may increase, suggesting a bacterial coinfection, pulse glucocorticoid administration, or acute rejection, and should be managed accordingly.

Elevated serum levels of D-dimer and I--Troponin were observed more frequently in those patients exhibiting more severe presentations, and should suggest the presence of microvascular thrombosis or disseminated intravascular coagulation, given the absence of clinically evident thromboembolic events (17). A lower serum albumin, and higher procalcitonin, CRP, and creatinine levels, should also be considered factors for worse prognosis (18, 9). Therefore, a recommendation is provided to test D-dimer, ferritin, procalcitonin, CRP, and I-Troponin levels in addition to routine biochemical determinations at the debut, and thereafter only in those patients not showing clinical improvement (20).

The vast majority of the hospitalized patients included in this study showed either uni- or bilateral patchy opacities or lobe condensations in the chest-X-ray (CXR), that may passed unnoticed in the first phase of the infection (10-30%), and became more evident later during the admission. Interestingly, an improvement in radiographic findings has been observed without specific antiviral treatment in 7-10 days after the beginning of the symptoms by Zhu et al. (15). Although they recommended seriated high-resolution chest computed tomographies to follow the course of the pneumonia, this strategy was strongly discouraged and not performed by most centers, as part of prevention efforts.

### COVID-19 treatment and drug interactions with immunosuppression

Optimal COVID-19 management is still under debate, and the therapeutic approach still lacks significant evidence. Apart from symptomatic support therapy, nor specific treatment neither best practice guidelines still exist for the management for KTRs with COVID-19. However, enhancement of personal protection precautions, early identification, and timely management of affected patients seems to be crucial, particularly in this special subgroup.

The indication for antiviral therapy is uncertain, and there are no approved drugs in this regard to date. A biphasic pharmacological approach to treating SARS-CoV-2 has been proposed. During the first 7-10 days from the onset of symptoms (phase-I) antiretrovirals (oseltamivir, ritonavir, darunavir, lopinavir, cobicistat), remdesivir or *chloroquine/hydroxychloroquine* may be considered. After this initial period (phase-II) immunosuppressive (calcineurin inhibitors) and immunomodulatory drugs (tozilizumab, sarilumab) may be of benefit.

Chloroquine/hydroxychloroquine (400 mg/12h for 24 hours and 200/12 h for 10 days): evidence supports its antiviral activity against the SARS *in vitro.* However, clinical evidence to recommend its use remains limited, and is based on the outcomes of a small series showing negativization of PCR-testing after 3 days of treatment (21). Given the better tolerability and safer adverse event profile, hydroxychloroquine should be recommended. Azythromycin in combination with hydroxychloroquine has been associated to a higher probability of PCR-negativization and has been used variably (Table-3).

**Table 3:** Summary of COVID-19 specific treatment, immunosuppression schedule adjustment, and ventilatory support requirements.

Author	COVID-19 treatment					Immunossupression schedule adjustment					Ventilatory support		
	Antiviral (%, agent)	HC (%)	TZ (%)	IV GC (%)	ATB (%)	Anti-Mb (%)	CNI (%)	m-TOR I (%)	GC (%)	AB (%, cause)	O_2_ Suppl (%)	Non-inv ventilat (%)	Mechanical Ventilat %)
Banerjee D, et al. (14)	14 (oseltamivir)	0	0	0	14	M:14, H:85	M: 57, R:14 H: 14	--	--	--	85	28	28
Alberici, et al, (7)	0	95	30	100	55	H: 100	H: 100	H: 100	H: 100	H: 100	65	10	10
CUKTP (1)	0	100	6	0	60	H: 92	M: 85, R: 7 H: 7	--	M: 100	H: 13	N/A	N/A	27
Zhang, et al, (24)	100 (oseltamivir or albidol)	0	0	20	20	H: 80	R: 100	--	R: 80	I: 20 (acute rejection)	N/A	N/A	N/A
Pereira, et al, (16)	3 (remdesivir)	91	21	24	66	R or H: 88	R or H: 18	--	R or H: 7	I: 2 (induction/acute rejection)	N/A	41	35
Akalin, et al, (13)	0	66	22 (16 leronlimab)	0	N/A	H: 86	H: 20	--	--	--	N/A	N/A	39
Zhu, et al, (15)	100 (umifenovir, oseltamivir, ribaviringanciclovir)	0	0	80	0	H: 90	R or H: 80		H:100	I: 70	100	30	0
Montagut-Marrahi, et al, (18)	100 (lopinavir / ritonavir, beta-INF, anakinra)	14	50	50	43	H: 100	--	H: 100	--	--	N/A	N/A	6
Nair, et al. (23)		100	0	30	100	H:100	H: 20, R:80	H: 100	H: 100	--	N/A	N/A	30

**HC**: hydroxychloroquine, **TZ**: tozilizumab; **IVGC**: intravenous glucocorticoids; **ATB**: broad spectrum antibiotics (including azythromicin); **Anti-Mb**: antimetabolite therapy; **CNI**: calcineurin inhibitors; **m-TOR I**: m-TOR inhibitors, **GC**: glucocorticoids; **AB**: monoclonal/polyclonal antibodies; **02 Suppl**: oxygen supplementation; **Non-inv**: non-invasive; **Ventilât**: ventilation: **INF**: interferon; **R**: reduced; **H**:held; **M**: maintained; **I**: increased

*Second generation antiretrovirals lopinavir/ritonavir* (200 mg/50 mg; 2 pills/12 hours; oral uptake for 14 days): Although a recent analysis failed to demonstrate significant benefit with lopinavir/ritonavir beyond the standard treatment for hospitalized adult patients with COVID-19, a higher proportion of patients experienced a clinical improvement, the interval to this improvement was shorter, and the patients were less likely to die from the disease or its complications (22). These data may support their consideration in the higher risk groups, including the KTRs. However, 71% of the patients included in one series showed improvement in lung infiltrates on imaging without any specific antiretroviral therapy after 7-10 days of admission (15).

*Remdesivir* (200 mg iv for 24 hours, and 100 mg iv/24 h for 9 days): this drug has shown proved efficacy in reducing the viral load and improving lung parameters in animal and *in vitro* models (incorporation to RNA chains) (19).

*Corticosteroids* (methylprednisolone 16 mg iv/24 h or equivalent prednisone): Given their anti-inflammatory effect, corticosteroids may be contraindicated in the phase-I of the disease, but conversely would have a role in phase-II, particularly in those patients exhibiting ARDS.

*Tozilizumab* (8mg/kg iv up to 800 mg) and *leronlimab:* these drugs would play a role in limiting the citokine release syndrome observed in phase-II, particularly in those exhibiting increasing requirements of oxygen or ventilatory support. A substantial decrease in the serum levels of Ik-6, and parallel clinical improvement have been documented after 1-3 doses of treatment (13).

*Ascorbic acid:* The multicentric clinical trial CITRIS-AL suggests a mortality decrease with its use in those patients with ARDS. No other evidence supporting it is available (19).

*Intravenous immunoglobulins* (1 g/Kg/d for 2 days or 400 mg/Kg/d for 5 days): They have been used in cases of severe pneumonia in a case-by-case basis. Their use is still under debate (19).

All of the above mentioned agents are being used in the context of clinical trials or as off-label medications on the basis of *in vitro* outcomes or biologic plausibility. Such medications can be used as per institutional protocols, but attention must be paid to interactions with immunosuppressive medications in KTRs. Two interactions of primary importance are the prolongation of the QT interval, and alterations in the metabolism of tacrolimus. Tacrolimus may prolong the QT interval itself in a dose-depending fashion, and its accumulation in the plasma may lead to fatal arrhythmia (torsades). Protease inhibitors (lopinavir/ritonavir) can dramatically increase tacrolimus serum levels by liver enzymatic inhibition. In addition, the combination of hydroxychloroquine and azythromicin may also increase the corrected QT--interval. Therefore, both drug combinations must be handled with extremely care when associated to tacrolimus. Conversely, no interactions have been described between tozilizumab and immunosuppresive drugs. Interestingly, no drug interactions have been reported among the series studied.

In addition, COVID-19 patients tend to be hypercoagulable, and prophylactic therapy with low molecular weight heparin or low-dose aspirin is strongly recommended. Apixaban has also be used for this purpose when D-dimer levels were higher than 3.0 microg/mL (19).

## Outpatient management

KTRs with mild symptoms may be managed via telemedicine as outpatients, but this strategy should be used in a case-by-case basis given the risks for rapid decompensation and relative insensiveness in the assessment of dyspnea and vital signs, thus resulting unuseful in high-risk patients. In fact, a dramatic 25% of patients managed with this approach in the series by Akalin et al. died at home (13).

For an outpatient approach the following criteria have to be met: lack of fever, no dyspnea, and ability to maintain close communication with the transplant team. The patient should be instructed for a 14-day period of self-isolation (or at least 7 days after resolution of the symptoms, whichever is longer). Fluid communication between patient and transplant team is crucial (every 48 hours) to assess not only for health, but for emotional status. Temperature should be checked twice daily and a close monitoring of progression or new development of symptoms is mandatory. A pulse-oximeter should be provided, to check oxygen saturation at least three times a day (12). An initial diagnosis is mandatory, and must include a blood sample test containing WBC count, lymphocyte count, CRP, basic metabolic panel, liver function test, and CXR. If the patient remains stable regarding symptoms, these tests should be repeated every 48-72 hours. The frequency of laboratory testing may return to baseline after clinical improvement. Conversely, if the laboratory tests worsen, then testing should be recommended in a shorter interval, and hospitalization should be strongly considered.

Criteria for hospitalization include one of the following: dyspnea, severe vomiting or diarrhea, inability to maintain oral hydration/medication uptake, confusion, persistent/worsening fever >38°C, oxygen saturation <94%, significant laboratory abnormalities (AKI, acute liver injury), two consecutive abnormal readings (>70 mg/L) for high sensitivity-CRP, or abnormal CXR (12). Even when the patient does not meet the previous criteria, but is thought to be at high-risk of decompensation, unnable to provide adequate self-care, or a close communication with the transplant team is not possible, hospital admission should be encouraged.

## Management of baseline immunosuppression regime

The management of immunosuppression in KTRs with COVID-19 is challenging, representing a delicate balance between infection control and allograft funtion. Maintaining or increasing the immunosuppressive load may impair viral clearance and facilitate infection progression, while holding or cessating it may precipitate an acute rejection. Firm evidence-based recommendations are not posible at this point due to a lack of sufficient experience, and therefore a wise case-by-case approach seems to be the most prudent management. Factors that would aid regarding this decision-making process include: age, comorbid conditions, severity of COVID-19 infection, time from transplantation, baseline graft function, prior history of rejection, and donor specific antibody panel (12, 13).

Decrease the doses of immunossuppressive drugs is based on the experience with other viral infections that may affect KTRs, and lower counts of CD3+, CD4+, CD8+ cells exhibited by these patients (13). Both situations may act in symbiosis to induce or worse a lymphocyte depletion. In addition, it has been suggested that patients receiving triple immunosuppression regimes present worse outcomes compared with those requiring manteinance with dual immunosuppression therapy alone when infected by COVID-19 (14, 23, 24). In the series conducted by the Columbia University Kidney Transplant Program, those patients requiring ICU admission, artificial ventilatory support, or those who died (15%) were receiving a triple immunossuppression regimen. This fact may reinforce the belief that an association must exist between the immunosuppresive load and predisposition to a more severe infection requiring hospitalization, ICU admission, or death (1). Interestingly, reducing immunossuppresive therapy for a short interval do not seem to lead to acute rejection in the short-term, in the light of the experience provided in this review. However, the long-term effect is still uncertain.

Therefore, mild symptomatic patients may be managed with the immunossuppression regimen unchanged. The manteinance of the immunosuppressive schedule may not compromise the antiviral immune effect in mild-to-moderate symptomatic patients either. In this way, the usual practice in patients with mild-to-moderate symptoms is to continue (preferable) or make reductions in the immunosuppresive drugs, according to worsening symptomatology. Definately, the no-modification approach may favor an increase in mortality rates for those patients requiring hospitalization, and thus an aggresive reduction of immunosuppression must be considered in cases of severe pneumonia or ARDS.

On the basis of experience with BK virus and CMV infections, a 50% dose reduction or complete cessation of antimetabolite drugs is appropriate (12). However, if the patient is worsening according to the laboratory findings, antimetabolites should be completely discontinued.

The appropriate time for reduction and the potential role of calcineurin inhibitors (CNI) during the hyperinflammatory phase of the disease remains unknown. The recommendation is to maintain tacrolimus and adjust to 4-6 ng/mL, based on the experience in treating BK virus nephropathy. However, some authors recommend withholding in cases of severe pneumonia. An argument favoring the use of Cyclosporin--A mantainance is based on on its ability to limit the viral proliferation in diverse coronaviruses (through its impact on ciclophylin A and B) (25). However, switching from Tacrolimus to Cyclosporin-A does not seem recommended. On the other hand, the increased levels of cytokines (IL-6 and others) and hyperactivated status (CCR6+, and Thl7) in CD4_+_ cells suggested for the phase-II, may be limited with the use of Tacrolimus (26, 27).

In regard to induction therapy, it is possible that lymphocyte-depleting antibodies would increase the risk for worsening, thus cessation in KTRs exhibiting severe symptoms seems prudent. However, a case-by-case decision based on the particular risk-benefit situation is encouraged. Betalacept administration should be deferred, and the patient should be converted to an alternative agent.

Finally, the optimal reintroduction of immunosuppressive agents after discharge remains unclear. Current estimates are that the viral shedding can occur for up to 14-37 days after symptomatic improvement. In addition, a probable association between the viral load, symptoms severity, and viral shedding has been suggested. Therefore, the number of variables makes difficult to adopt a standardized interval regarding the reintroduction of immunosuppression. Nevertheless, to differ reintroduction at least for 2 weeks after symptoms improvement is recommended, recognizing the increased risk for allograft rejection in the interim.

## CONCLUSIONS

The sudden spreading of COVID-19 across the globe has brought uncertanty regarding the diagnosis and treatment of the disease. Although general understanding is improving, information about select patient subgroups, such as KTRs, remains limited and deserve special consideration. The ideal treatment for KTRs with SARS-CoV-2 infection remains unclear, and the answers regarding its optimal management still rely on expert opinion. Although many of the patients included in this review experienced a favorable outcome, the small cohort and varied therapy makes it difficult to draw any meaningful conclusion beyond that of short-term safety and tolerability of the currently available protocols. Long-term follow-up is required to better understand the prognosis and sequelae of COVID-19 in KTRs.
